# The Oenological Potential of *Hanseniaspora uvarum* in Simultaneous and Sequential Co-fermentation with *Saccharomyces cerevisiae* for Industrial Wine Production

**DOI:** 10.3389/fmicb.2016.00670

**Published:** 2016-05-09

**Authors:** Mariana Tristezza, Maria Tufariello, Vittorio Capozzi, Giuseppe Spano, Giovanni Mita, Francesco Grieco

**Affiliations:** ^1^Consiglio Nazionale delle Ricerche–Institute of Sciences of Food ProductionLecce, Italy; ^2^Department of the Sciences of Agriculture, Food and Environment, University of FoggiaFoggia, Italy

**Keywords:** oenological non-*Saccharomyces*, wine alcoholic fermentation, *Hanseniaspora uvarum*, *Saccharomyces cerevisiae*, mixed fermentations, starter multi-strains, co-inoculation, sequential inoculation

## Abstract

In oenology, the utilization of mixed starter cultures composed by *Saccharomyces* and non-*Saccharomyces* yeasts is an approach of growing importance for winemakers in order to enhance sensory quality and complexity of the final product without compromising the general quality and safety of the oenological products. In fact, several non*-Saccharomyces* yeasts are already commercialized as oenological starter cultures to be used in combination with *Saccharomyces cerevisiae*, while several others are the subject of various studies to evaluate their application. Our aim, in this study was to assess, for the first time, the oenological potential of *H. uvarum* in mixed cultures (co-inoculation) and sequential inoculation with *S. cerevisiae* for industrial wine production. Three previously characterized *H. uvarum* strains were separately used as multi-starter together with an autochthonous *S. cerevisiae* starter culture in lab-scale micro-vinification trials. On the basis of microbial development, fermentation kinetics and secondary compounds formation, the strain *H. uvarum* ITEM8795 was further selected and it was co- and sequentially inoculated, jointly with the *S. cerevisiae* starter, in a pilot scale wine production. The fermentation course and the quality of final product indicated that the co-inoculation was the better performing modality of inoculum. The above results were finally validated by performing an industrial scale vinification The mixed starter was able to successfully dominate the different stages of the fermentation process and the *H. uvarum* strain ITEM8795 contributed to increasing the wine organoleptic quality and to simultaneously reduce the volatile acidity. At the best of our knowledge, the present report is the first study regarding the utilization of a selected *H. uvarum* strain in multi-starter inoculation with *S. cerevisiae* for the industrial production of a wine. In addition, we demonstrated, at an industrial scale, the importance of non-*Saccharomyces* in the design of tailored starter cultures for typical wines.

## Introduction

Fermentation associated with wine production represents complex biological processes denoted by several biochemical interactions between grape must and different micro-organisms such as fungi, yeasts and bacteria (Fleet, 2003). In particular, yeasts play a fundamental role, since they carry out the alcoholic fermentation (AF), i.e., the conversion of sugars to ethanol and CO_2,_but they also determine the wine organoleptic properties by producing and secreting into the fermenting must several secondary metabolites (Lambrechts and Pretorius, 2000; Fleet, 2003; Romano et al., 2003; Jolly et al., 2006). The AF is initially promoted by the action of a heterogeneous consortium of yeasts belonging to different non*-Saccharomyces* species usually characterized by a low fermentative power (Heard and Fleet, 1985), while its final step is under the control of alcohol-tolerant *Saccharomyces cerevisiae* strains (Fleet and Heard, 1993). The function of non*-Saccharomyces* species throughout the AF is very significant, since they strongly contribute in determining the wine chemical composition. Autochthonous yeasts provide distinctive regional features to wines (Romano et al., 2003; Fleet, 2008; Ciani et al., 2010; Medina et al., 2013; Garofalo et al., 2015) thus advising their use as commercial starter cultures in order to differentiate wine productions. Some non*-Saccharomyces* yeasts are already commercialized as oenological starter cultures (e.g., *Torulaspora delbrueckii, Metschnikowia pulcherrima, Pichia kluyveri, Lachancea thermotolerans*) to be used in combination with *Saccharomyces cerevisiae* (Lu et al., 2015), while several others are the subject of various studies (e.g., *Hanseniaspora uvarum, Starmerella bacillaris*) (Masneuf-Pomarede et al., 2016).

The apiculate yeast *Hanseniaspora uvarum* (anamorph *Kloeckera apiculata*) is one of the yeast species more represented onto grape berries and they prevail in the first steps of spontaneous AF (Fleet and Heard, 1993). This yeast species is important in the production of volatile compounds in wine and the general chemical composition of wines made by *Hanseniaspora* spp.*/S. cerevisiae* combinations may differ from reference wines produced with pure culture of *S. cerevisiae* (Zironi et al., 1993; Erten, 2002; Ciani et al., 2006; Gil et al., 2006). Previous reports indicated that several *H. uvarum* physiological properties of oenological interest are strain-dependent characters, such as ethanol production (Caridi and Ramondino, 1999), the volatile acidity associated with fermentation (Romano et al., 1992; Ciani and Maccarelli, 1998) and, most of all, the production of primary metabolites (i.e., glycerol, acetaldehyde) and secondary metabolites, such as ethyl acetate and hydrogen sulfide (Romano et al., 1997).

During a recent investigation, we have studied the oenological properties of 9 different *H. uvarum* strains isolated during the first 24 h of the spontaneous fermentation of Negroamaro grape must (De Benedictis et al., 2011). The chemical analysis of fermented must showed that all the strains produced low amounts of hydrogen sulfide and acetic acid, showing fructophilic character and relevant glycerol production. Analysis of volatile compounds indicated that in particular one strain, *H. uvarum* ITEM8795, could potentially enhance taste and flavor of wines, thus indicating its possible utilization for the formulation of mixed and/or sequential starters together with *S. cerevisiae* strains.

Indeed, for several non-*Saccharomyces* yeasts species has been demonstrated that they contribute to the analytical composition and the sensorial characteristics of wine, increasing the interest in the industrial application of apiculate yeasts (Pérez-Coello et al., 1999; Domizio et al., 2007; Fleet, 2008; Viana et al., 2008; Capozzi et al., 2015). In fact, the addition of non-*Saccharomyces* yeast species as part of mixed starter formulations, together with *S. cerevisiae* (and of malolactic bacteria), has been recently indicated as a way of mimic the spontaneous fermentations (Mendoza and Farías, 2010; Suzzi et al., 2012), conferring a particular aroma and characteristics to wines (Ciani et al., 2010; Comitini et al., 2011; Suárez-Lepe and Morata, 2012) without increasing/reducing the risks for wine quality and safety often associated with uncontrolled vinifications (Spano et al., 2010; Capozzi and Spano, 2011; Tristezza et al., 2013).

On the above basis, the aim of the present study was to assess the fermentation performances and interactions of mixed cultures and sequential inoculation of *H. uvarum* and *S. cerevisiae*. Data about microbial development, fermentation kinetics and secondary compound formation in lab-scale micro-vinification trials were further confirmed by utilization of the above mixed starter in pilot- and industrial-scale production of Negroamaro wine. At the best of our knowledge, the present investigation is the first report about the utilization of selected strain of *H. uvarum* in simultaneous and sequential co-fermentation with *S. cerevisiae* from micro-vinification up to the industrial scale in the production of a typical red wine.

## Materials and methods

### Yeast strains

Yeast strains used in the present study are deposited in Agro-Food Microbial Culture Collection of ISPA (http://www.ispacnr.it/collezioni-microbiche/). The *Saccharomyces cerevisiae* strain ITEM6920 (S) and the *Hanseniaspora uvarum* strains ITEM8795 (H1), ITEM8797 (H2), ITEM8799 (H3) have been previously isolated from spontaneous fermentation of Negroamaro grapes (De Benedictis et al., 2011; Tristezza et al., 2012). All the strains had been previously identified and characterized for their oenological properties, and in particular, the *S. cerevisiae* strain ITEM6920 has been already used as starter culture for the industrial production of Negroamaro wine (De Benedictis et al., 2011; Tristezza et al., 2012). The yeast strains were sub-cultured on YEPD (10 g/L yeast extract, 20 g/L peptone, 20 g/L glucose, 20 g/L agar) and maintained at −80°C in glycerol 50% (Bleve et al., 2011). Screening of Killer-Sensitive pattern (killer, sensitive and neutral phenotypes) was carried out as described by Jacobs et al. (1988).

### Microfermentations

Fermentation tests were carried out at 25°C in 500 mL flask containing 450 mL of Negroamaro grape must (205 g/L sugars, pH 3.44, assimilable nitrogen concentration 142.14 g/L) added with 20 mg/L of potassium metabisulphite. The must was clarified by centrifugation (10 min at 8000 g) and then sterilized by membrane filtration through Millipore system (0.45 μm membrane). Each flask was inoculated with the required concentration of a yeast pre-culture in the same must (48 h at 25°C), as previously described (Grieco et al., 2011).

The *H. uvarum* strains were inoculated at 10^7^ CFU/mL, while the *S. cerevisiae* strain ITEM6920 was inoculated, in a preliminary test, at three different concentrations: 10^7^, 10^5^, and 10^3^ CFU/mL, in order to reach, respectively, the inoculation ratios *H. uvarum*:*S. cerevisiae* of 1:1, 100:1, and 10,000:1. Each *H. uvarum* strain was inoculated in combination with the *S. cerevisiae* strain in two different timings: simultaneous inoculum (SM) and sequential inoculum (SQ). In the case of SQ, *S. cerevisiae* was inoculated after *H. uvarum*, when alcohol content reach 5% v/v. Fermentation kinetics were monitored daily by gravimetric determinations until constant weight and then the samples were stored at −20°C until analysis. Each fermentation experiment was carried out in duplicate. A pure culture of the *S. cerevisiae* strain was also inoculated as positive control, as well as a non-inoculated must was used as negative control.

### Pilot-scale vinification

The selected strains were tested in pilot-scale fermentation trials. The vinification was carried out in an experimental cellar using sterile stainless steel 100-L vessels (Grieco et al., 2011) by inoculating 90 L of Negroamaro must (240 g/L of total sugars, 232 mg/L of yeast assimilable nitrogen, pH 3.52, added with 20 g/hL of potassium metabisulphite) with 10^7^ cell/mL of *H. uvarum* and 10^5^ cell/mL of *S. cerevisiae*, both in simultaneous and sequential approach. *S. cerevisiae* inoculated alone was used as control. The kinetics of the alcoholic fermentation process was monitored daily measuring the density. Samples of must and wines were collected as single replicate and stored at −20°C for further analyses.

### Industrial vinification

Industrial fermentation was carried out in a 100,000 L stainless steel vessel. To start must fermentation on large scale, the initial inocula were prepared, transported to the winery and used as starters (Tristezza et al., 2012). The mixed starters cultures of *Hanseniaspora uvarum* strains ITEM8795 and *S. cerevisiae* ITEM6920, respectively corresponding to 7 × 10^12^ CFU/hL and 7 × 10^10^ CFU/hL, were mixed with 300 kg of and let for 6 h at room temperature. After this period, the yeast-must mixture was added to 7 tons of Negroamaro must (212.8 g/L of total sugars, pH 3.33, yeast assimilable nitrogen 158.8 g/L, added with 20 g/hL of potassium metabisulphite). The alcoholic fermentation process was carried out at 25°C and its kinetics was daily monitored by measuring the reducing sugars concentration and density. Samples of must and wines were collected as single replicate for further chemical and microbiological analysis.

### Differential enumeration of yeast populations

In order to determine microbial growth, must and wine samples were collected over the fermentation processes. Serial dilutions of each sample were spread on WL Nutrient Agar (WLN medium, Oxoid, UK) and Lysine Agar (LA medium, Oxoid, UK). LA medium was used for the enumeration of non-*Saccharomyces* yeast population while WLN was used for differential enumeration of total yeast population (Pallmann et al., 2001). The identification of *H. uvarum* and *S. cerevisiae* was carried out by performing a molecular assay. Yeast colonies, showing a typical phenotype, were selected from WLN plates, and their genomic DNA was extracted according to Tristezza et al. (2009). RAPD pattern of *H. uvarum* were performed according to De Benedictis et al. (2011), while interdelta profiles of *S. cerevisiae* were analyzed as described by Tristezza et al. (2012).

### Analytical determinations

The main chemical parameters of wines and musts were analyzed by Fourier Transform Infrared Spectroscopy (FTIR), employing the WineScan Flex (FOSS Analytical, DK). Samples were centrifuged at 8000 rpm for 10 min and then analyzed following the supplier's instructions. Acetaldehyde, ethyl acetate and acetoin were determined by gas-chromatography according to Mallouchos et al. (2003). The internal standard solution used was 4-methyl-1-pentanol. Free volatile compounds were extracted by solid phase extraction method (SPE) and analyzed by gas chromatography–mass spectrometry (GC–MS) as previously described (Tufariello et al., 2012). The Odor Activity Values (OAVs) were calculated according to Capone et al. (2013). To evaluate the contribution of a volatile compound to the aroma, the Odor Activity Value (OAV) was calculated as the ratio between the concentration of each compound and the perception threshold in a specified matrix reported in literature (Swiegers et al., 2005). An aromatic series was defined as a group of volatile compounds with similar aroma descriptors (i.e., floral, sweet, fruity, spicy, green, fatty). The value of each aromatic series was calculated as the sum of the OAVs of the compounds that integrate it. Fermentation rate (FR), fermentation purity (FP), and alcohol yield coefficient (AYC) were calculated according to Tristezza et al. (2012).

### Statistical treatment of data

Statistical data processing was performed using the free software package PAST (Hammer et al., 2001).

## Results

### Microfermentations

In a preliminary experiment we have studied the growth kinetics of the *H. uvarum/S. cerevisiae* mixed cultures (data not shown). The growth dynamics of the *H. uvarum* strains were comparable in the tests when the inoculum ratio were equivalent to 100:1 and 10.000:1, i.e., the *S. cerevisiae* starter inoculated at 10^5^ and 10^3^ CFU/mL, which reached a concentration of 10^7^ CFU/mL respectively after 7 and 15 days. However, when the *S. cerevisiae* strain was inoculated at 10^7^ CFU/mL (inoculum ratio of 1:1) the non-*Saccharomyces* cell concentration declined after 5 days (data not shown). For these reasons, the inoculum amount chosen for further experiments were 10^7^ CFU/mL for *H. uvarum* strains and 10^5^ CFU /mL for the *S. cerevisiae* starter (ratio 100:1). The fermentation kinetics of mixed cultures in micro-vinification trials are reported as Supplementary Data (Supplementary Figure 1). Two different temporal approaches were tested: a simultaneous inoculation of the two species and a sequential inoculation with a delay of 2 days in the addition of *S. cerevisiae* after *H. uvarum*. The time courses of simultaneous trials were comparable to those of the *S. cerevisiae* pure culture. The sequential trials presented a decrement of the initial fermentation rate that was higher for ITEM8797 (H2) and ITEM8799 (H3). Nonetheless, all trials gave complete fermentations in 14 days.

**Figure 1:**
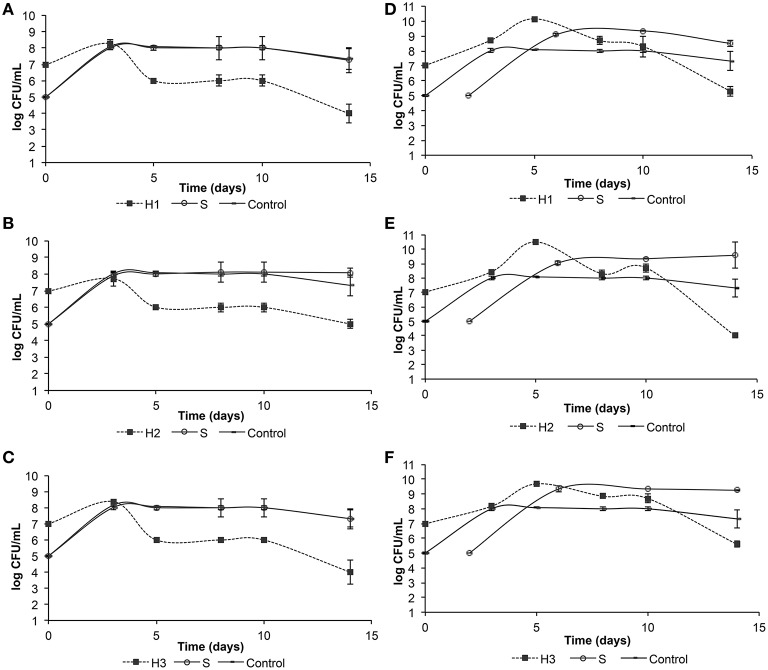
**Evolution of yeast populations in micro-vinification conditions with simultaneous inoculation (A, *H. uvarum* ITEM 8795 + *S. cerevisiae* ITEM 6920; B, *H. uvarum* ITEM 8797 + *S. cerevisiae* ITEM 6920; C, *H. uvarum* ITEM 8799 + *S. cerevisiae* ITEM 6920) and sequential inoculation (D, *H. uvarum* ITEM 8795 + *S. cerevisiae* ITEM 6920; E, *H. uvarum* ITEM 8797 + *S. cerevisiae* ITEM 6920; F, *H. uvarum* ITEM 8799 + *S. cerevisiae* ITEM 6920)**. Values are mean of two independent duplicates.

The microbial population dynamics of the mixed fermentations are shown in Figure 1. In all simultaneous trials (Figures 1A–C), both strains reached a maximum population (around 10^8^ CFU/mL) after 72 h. Viable counts of *S. cerevisiae* kept stable at 10^8^ CFU/mL until the 10th day of fermentation. Then, in mixed fermentation with ITEM8795 (H1) (Figure 1A) and H3 (Figure 1C), cells counts slightly decrease at 10^7^ CFU/mL. From the 3rd to the 5th day, all the three *H. uvarum* strains decreased in viable counts at 10^6^ CFU/mL and kept stable until the 10th day. At the end of the fermentations, the number of viable cells of H2 was 10^5^ CFU/mL, whereas for H1 and H3 it was up to 10^4^ CFU/mL. In the three sequential trials (Figures 1D–F), *H. uvarum* reached a maximum (10^10^ CFU/mL) in 5 days and then decreased at 10^9^ CFU/mL. By the end of the fermentations, viable counts were 10^5^ CFU/mL for H1, 10^4^ CFU/mL for H2 and 10^6^ CFU/mL for H3. The strain of *S. cerevisiae* showed a similar trend in the three trials: reached a maximum (10^9^ CFU/mL) 3 days after inoculation and kept constant until the end of the fermentations. The pure culture of *S. cerevisiae* ITEM6920 (S) used as control reached a maximum population (10^8^ CFU/mL) in 3 days and kept constant until the 10th day post-inoculation. By the 14th day, viable counts were 10^7^ CFU/mL. Moreover, the tests carried out to assess the killer toxin activity excluded any cross inhibition between the *H. uvarum* and *S. cerevisiae* strains under study (data not show).

The oenological parameters of the mixed fermentations and the pure culture are shown in Table 1. As expected considering the fermentation kinetics, all the trials finished the fermentation leaving in the must less than 3 g/L of residual sugars. The highest ethanol concentration was determined in the pure culture of *S. cerevisiae* while all the mixed fermentations reached a lower ethanol concentration ranging from 11.92 to 12.19 mL/100 mL. On the other hand, the production of glycerol was greater (6.15–6.33 g/L) in mixed fermentations than in the control (5.24 g/L). The activity of *H. uvarum* did not increase volatile acidity; in fact the co-inoculated trials had a volatile acidity concentration statistically lower than the control. Fermentation purity (ratio between volatile acidity and ethanol produced) were also very low (0.03) for all samples, highlighting the good oenological performance of these mixed starters.

**Table 1:** **Concentration of major chemical compounds in fermented musts obtained with mixed cultures of *H. uvarum* /*S. cerevisiae* strains and with the pure culture of *S. cerevisiae* as control**.

	**Simultaneous**			**Sequential**			**Control**
	**H1+S**	**H2+S**	**H3+S**	**H1+S**	**H2+S**	**H3+S**	**S**
Alcohol (mL/100 mL)	12.14±0.114	12.05±0.038	12.19±0.104	11.98±0.021	11.92±0.028	11.96±0.007	12.33±0.007
Residual sugars (g/L)	2.09±0.047	2.15±0.153	2.19±0.113	2.13±0.092	2.25±0.212	2.17±0.099	2.18±0.120
Total acidity (g/L)	6.30±0.029	6.43±0.087	6.46±0.083	6.35±0.064	6.52±0.014	6.49±0.028	6.34±0.035
Volatile acidity (g/L)	0.34±0.000	0.37±0.008	0.34±0.015	0.41±0.014	0.40±0.007	0.42±0.014	0.41±0.000
pH	3.34±0.005	3.33±0.014	3.33±0.008	3.32±0.007	3.31±0.007	3.31±0.000	3.29±0.000
Tartaric acid (g/L)	1.87±0.029	1.80±0.068	1.92±0.143	1.63±0.028	1.66±0.042	2.11±0.092	1.73±0.078
Glycerol (g/L)	6.32±0.151	6.21±0.266	6.32±0.243	6.15±0.042	6.33±0.085	6.22±0.007	5.24±0.078
Acetaldehyde (mg/L)	20.05±0.451	19.96±0.382	20.32±0.297	21.4±0.600	21.95±0.190	22.41±0.216	24.22±0.164
Ethyl acetate (mg/L)	84.78±0.753	96.57±0.822	98.33±1.254	104.22±2.660	107.53±3.918	106.88±2.674	44.53±0.980
Acetoin (mg/L)	11.24±1.045	12.33±1.562	12.89±1.664	12.77±1.331	13.05±1.258	12.87±1.744	4.25±0.563

*Values are the mean of two injections of each replicate; the standard deviation values (±) are indicated; n.d. not detectable*.

The capacity to produce a number of volatile compounds susceptible to be involved in the wine flavor formation (acetaldehyde, ethyl acetate and acetoin) was also assessed in mixed fermentations (Table 1). The acetaldehyde, one of the most important carbonyl compounds synthetized all through the alcoholic fermentation, was detected, within the range between 11.24 mg/L (H1+S) and 12.89 mg/L (H3+S) for simultaneous inoculation and within the range between 12.77 mg/L (H1+S) and 13.05 mg/L (H2+S) for sequential inoculation. The free acetaldehyde has a dual role in flavor formation; at moderate concentrations it contributes to fruity flavors, while high levels (>200 mg/L) suppress the aroma in wines. The ethyl acetate was identified in concentrations ranging from 84.78 mg/L (simultaneous inoculum with H1+S) to 107.53 mg/L (sequential inoculum with H2+S). Ethyl acetate may add pleasurable, fruity aroma to the general wine bouquet at low concentrations, whereas it appreciably affect the final aroma at a content higher than 150 mg/L (Lambrechts and Pretorius, 2000). The acetoin (3-hydroxy-2-butanone) odor threshold is relatively high (150 mg/L) and, consequently, its sensory meaning for the global aroma is nearly irrelevant. All the *H. uvarum* strains under study produced a low amount of the above compound, either in the simultaneous and in the sequential inoculum, within the range of 11.24 mg/L (simultaneous inoculum with H1+S) to 13.05 mg/L (sequential inoculum with H2+S).

**Figure 2:**
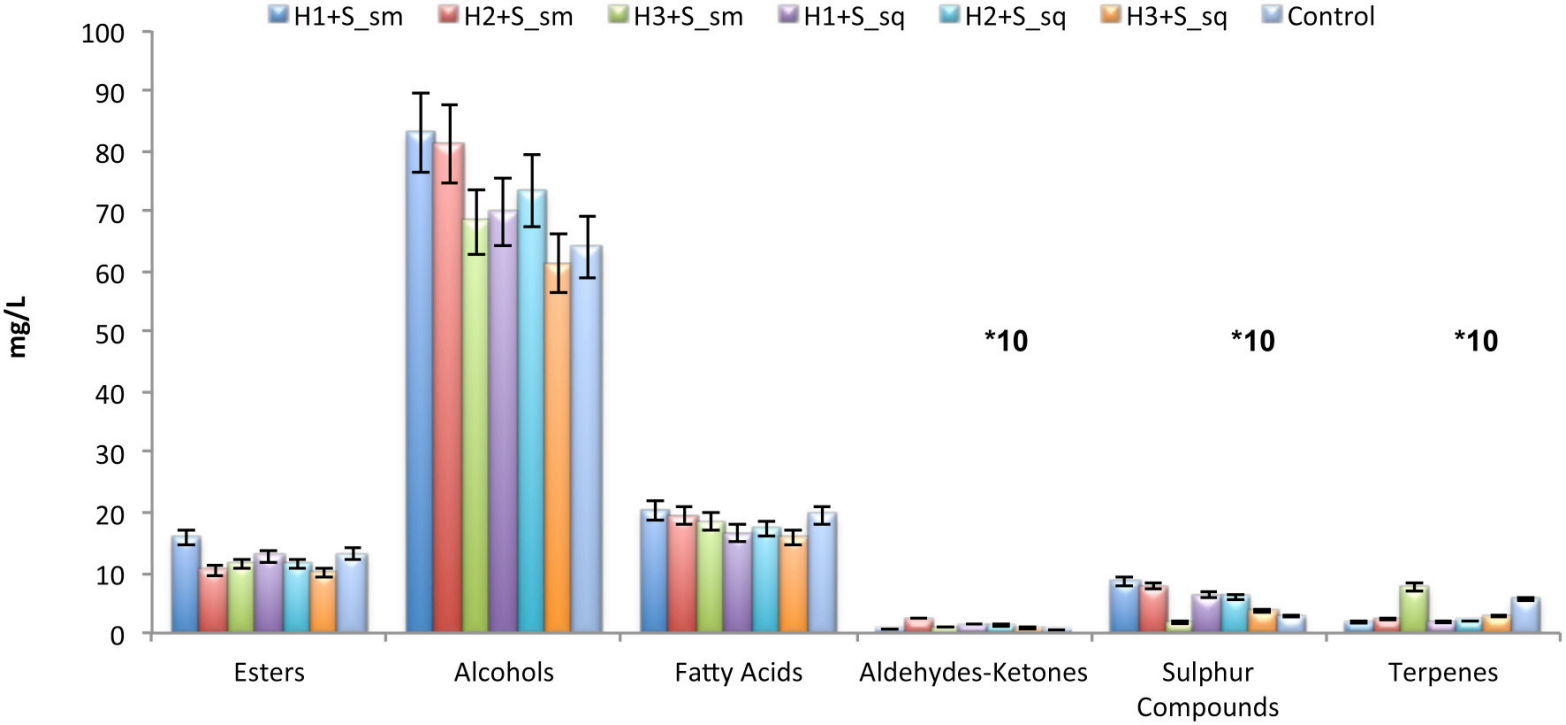
**Volatile composition of wines obtained in micro-vinification conditions with simultaneous (sm) and sequential (sq) inoculation**. H1, *H. uvarum* ITEM 8795; H2, *H. uvarum* ITEM 8797; H3, *H. uvarum* ITEM 8799; S, *S. cerevisiae* ITEM 6920. The pure culture of *S. cerevisiae* ITEM 6920 (S) was used as control. The concentrations of the aldeydes-ketones, sulfur compounds and terpenes have been multiplied by a factor of 10. Error bars indicate standard deviation.

To determine the effect of co-inoculums and sequential yeasts on the final composition of wine, experimental wines were analyzed by gas chromatography. The comparison of the results obtained is shown in Figure 2. Generally, simultaneous trials produced a higher amount of volatile compounds, esters, alcohols and terpenes. The co-inoculated couple H1+S presented the highest formation of esters (15.7 mg/L), alcohols (83 mg/L), and organic acids (20.4 mg/L). Also the co-inoculated couple H2+S presented high concentrations of alcohols (81.2 mg/L) and organic acids (19.4 mg/L) but lower amounts of esters (10.3 mg/L).

Analysis of these compounds provides a simply way of measuring the ability of different strains to produce wines with different profiles, since the main difference among wines inoculated with different yeast strains lies in the concentration of aromatic compounds rather than in the type of metabolite produced (Romano et al., 1997).

**Figure 3:**
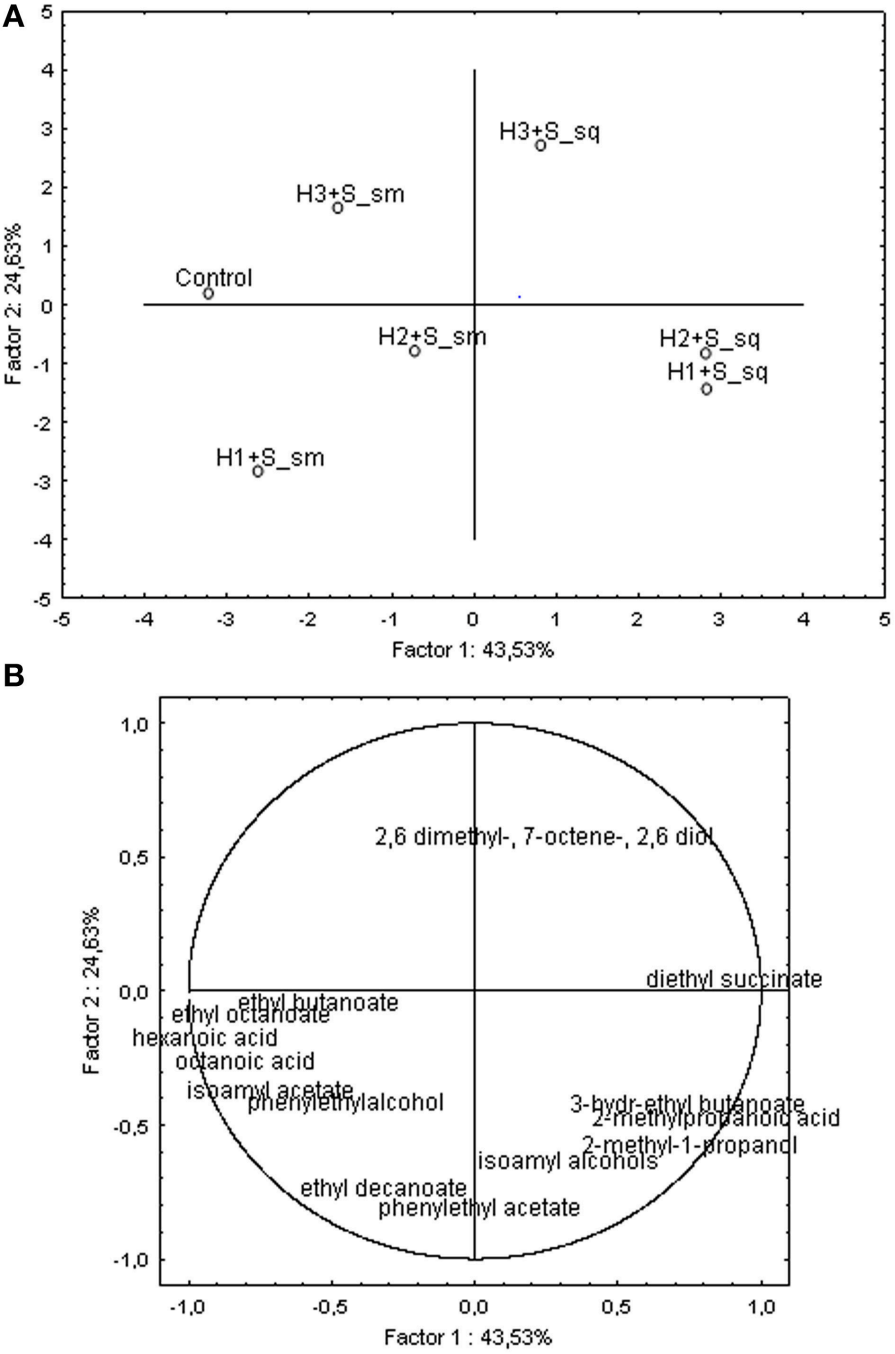
**Two-dimensional principal component analysis (PCA)**. Scores plot **(A)** for wines obtained in micro-vinification conditions and loading plot **(B)** for volatiles higher than odor threesold. Simultaneous (sm) and sequential (sq) inoculation. H1, *H. uvarum* ITEM 8795; H2, *H. uvarum* ITEM 8797; H3, *H. uvarum* ITEM 8799; S, *S. cerevisiae* ITEM 6920.

PCA was used to identify the specific volatile compounds best discriminating among the wines produced by co-inoculum (i.e., H1+S sm, H2+S sm, H3+S sm) and sequential (i.e., H1+S sq, H2+S sq, H3+S sq) techniques studied (Figure 3). PCA was initially applied to the concentrations of the volatile compounds in concentrations higher than their odor threshold are mainly considered as aroma-contributing substances (Gómez-Mínguez et al., 2007). The two principal components, PC1 and PC2, accounted for 68.16% of the total variance (43.53 and 24.63%, respectively). The second dimension (24.63% of explained variance) discriminates these two techniques studied, simultaneous (sm) and sequential (sq) inoculum.

However, samples H2+S sm, was associated to negative PC1(34% of explained variance), that discriminates H2+S sm and H1+S sm from the two other samples, Control S and H3+S sm for the high content, besides other variables, of ethyl octanoate, ethyl butanoate, terpens responsible of floral and fruity notes. The sample H1+S sm clustered at negative PC1 and PC2 scores thus showed relatively high correlations mainly with hexanoic and octanoic acids, phenylethylalcohol and isoamyl acetate. Samples H1+S sq and H2+S sq that cluster at positive PC1 scores scored high relative correlations with diethyl succinate, 2-methyl-propanoic acid, 2-methyl-1-propanol and 3-hydroxy-ethyl butanoate. Finally, H3+S sq that clusters at positive PC2 associated to 2,6-dimethyl-7-octen-2,6 diol. In conclusion, it was found that cultures in co-inoculum positively influenced the production of different classes of volatiles, terpenes, esters, acids and alcohols. In particular H1+S sm was characterized by a higher yield of most volatile components that influence positively aroma bouquet, such as isoamyl acetate, ethyl octanoate, phenylethyl acetate (fruity notes), phenylethylalcohol (floral notes), hexanoic and octanoic acids.

### Pilot-scale vinification

In reason of the performances in the micro-vinification trials, the *Hanseniaspora uvarum* strain ITEM8795 (H1) was selected to be tested in pilot-scale fermentations, both in simultaneous and sequential approaches, with *S. cerevisiae* ITEM6920. An identical amount (90 L) of the same Negroamaro must was inoculated with *S. cerevisiae* alone as control. Fermentation rate was higher for the two mixed starter fermentations than for that inoculated with the *S. cerevisiae* pure culture. Co-inoculation of *H. uvarum* and *S. cerevisiae* lead to a complete fermentation after 6 days (not shown). The three fermentations resulted in a different profile of sugars consumption (Supplementary Figure 2 in the Supplementary Data section). As can be observed, the simultaneous inoculation showed a good fermentation performance which led to a complete consumption of glucose in 4 days and fructose in 8 days. In addition, the sequential inoculation showed good fermentation properties with a complete consumption of glucose in 6 days and 5.7 g/L of residual fructose by the 12th day. On the contrary, the pure culture of *S. cerevisiae* showed a less efficient profile of sugar consumption with a complete consumption of glucose in the 8th day of fermentation and a residual fructose of 7.2 g/L at day 12. The development of yeast populations during the three fermentations is shown in Supplementary Figure 3 (Supplementary Data). The *H. uvarum* strain reached its maximum (10^8^ CFU/mL) at the 2nd day, both in simultaneous and sequential trials; then, viable cells counts decreased at 10^3^ CFU/mL (day 4th), subsequently at 10^2^ CFU/mL, by the 6th day, and kept stable until the 11th day.

Viable cells counts of *S. cerevisiae* in simultaneous fermentation reached their maximum (10^8^ CFU/mL) at the 2nd day and then slightly decreased at 10^7^ CFU/mL until the end of fermentation (11th day). In sequential inoculation, *S. cerevisiae* reached a maximum population (10^9^ CFU/mL) in 4 days and then gradually decreased. By the 11th day, viable counts were 10^7^ CFU/mL. The pure culture of *S. cerevisiae* used as control showed a similar trend: reached a maximum (10^9^ CFU/mL) 4 days after inoculation and constantly decreased to 10^7^ CFU/mL until the end of the fermentations.

The analytical SPE/GC–MS method, used in this work for the analysis of wine samples, allowed the correct identification and quantification of 45 volatile compounds (Table 2). All the volatile compounds were grouped according to the belonging class (esters; aldehydes/ketons; alcohols; phenols; lactones; terpenes; acids). For each compound, the odor threshold (OTH) and the sensory odor descriptor were also reported. With respect to esters, it is important to highlight that wine produced by co-inoculation contained high concentrations of ethyl butyrate, isoamyl acetate, ethyl hexanoate responsible of fruity notes. On the contrary, concentrations of diethyl succinate, ethyl 9-decenoate, 2-phenylethyl acetate and diethyl malate were significantly lower in wines from co-inoculation assays. Ethyl esters are mainly synthesized by yeast starting from grape precursors and by ethanolysis of acylCoA that is formed during fatty acid synthesis or degradation.

**Table 2:** **Concentration of major volatile compounds in fermented musts obtained with the mixed starter *H. uvarum*/*S. cerevisiae* used in sequential or simultaneous inoculum**.

**Volatile compounds**	**Odor threshold (μg/L)^a^**	**Odor descriptor**	**Odorant series^b^**	**H1+S Simultaneous**	**H1+S Sequential**	**S**
				**μg/L**	**μg/L**	**μg/L**
**ESTERS**						
Ethyl butyrate	20 (a)	Fruity	1	425 ± 77	319 ± 84	386 ± 5
Isoamyl acetate	30(c)	Banana	1	2535 ± 1	2239 ± 140	2235 ± 49
Ethyl hexanoate	14 (b)		1	645 ± 118	561 ± 26	510 ± 17
Ethyl lactate	154,636 (c)	Acid. medicine	6	1028 ± 459	720 ± 104	1006 ± 32
Ethyl caprilate (octanoate)	5 (b)	Sweet. fruity	1.4	548 ± 111	573 ± 74	406 ± 42
3-hydroxy. ethyl butyrate	20,000 (b)	Caramel. Toasted	4	52 ± 34	65 ± 10	52 ± 2
Ethyl (decanoate) caprate	200 (c)	Sweet. fruity	1.4	219 ± 56	252 ± 71	188 ± 27
Diethyl succinate	200,000 (b)	Vinous	7	3735 ± 1820	4216 ± 1820	3851 ± 212
Ethyl 9 decenoate	14,100			200 ± 46	234 ± 88	102 ± 6
2-phenyl ethyl acetate	250 (a)	Floral	2	598 ± 93	696 ± 125	517 ± 65
Diethyl malate	760,000 (b)	Over-ripe. peach. cut grass	1	340 ± 164	525 ± 310	291 ± 31
4 hydroxy-3 methoxy benzoic acid ethyl ester (ethyl vanillate)	990 (b)	Sweet. vanillin	4.5	nd	4855 ± 21	Nd
Ethyl monosuccinate	1,000,000 (c)	Caramel. coffee	4	5648 ± 318	6052 ± 552	8476 ± 311
TOTAL				15,975 ± 3296	21,306 ± 3405	18,021 ± 799
**CARBONYL COMPOUNDS**						
Acetaldehyde	500 (a)	Pungent. ripe apple	1.6	269 ± 21	155 ± 65	125 ± 7
Acetoin	150,000			538 ± 192	nd	544 ± 26
Furfural	14,100 (c)			nd	nd	nd
Benzaldehyde	350 (c)	Sweet. fruity	1.4	94 ± 35	70 ± 6	58 ± 6
TOTAL				901 ± 248	224 ± 71	728 ± 38
**ALCOHOLS**						
1-propanol	830 (b)		1.6	312 ± 33	nd	211 ±17
Isobutanol	40,000 (b)		3.6	966 ± 566	701 ± 362	1427 ± 13
1-butanol	150,000 (b)	Medicinal. phenolic	6	109 ± 9	nd	178 ± 7
Isoamyl alcohol	30,000 (a)	Burnt. alcohol	4.6	14,785 ± 3772	13,968 ± 3525	15,754 ± 201
3-methyl-1-pentanol	50,000 (c)	Vinous. herbaceous. cacao	1.3.7	124 ± 43	118 ± 21	142 ± 7
1-hexanol	8000 (a)	Flower. green. cut grass	2.3	492 ± 196	491 ± 220	776 ± 22
(E)-3-hexen-1-ol				55 ± 31	79 ± 14	81 ± 5
(Z)-3-hexen-1-ol	400 (a)		3	66 ± 21	80 ± 2	56 ± 14
2.3-butanediol *(levo)*	15,0000 (b)	Fruity	1	2712 ± 1238	nd	1063 ± 48
2.3-butanediol *(meso)*		fruity		820 ± 79	nd	296 ± 30
Methionol	1000 (a)	Cooked vegetable	7	196 ± 82	203 ± 0	261 ± 8
Benzylalcohol	200,000 (b)	Sweet. fruity	1.4	190 ± 20	184 ± 30	179 ± 16
Phenylethylalcohol	10,000 (a)	Floral. roses	2	11,577 ± 2399	12,962 ± 3194	13,760 ± 1186
TOTAL				31,939 ± 8488	28,786 ± 7367	34,184 ± 1574
**PHENOLS**						
Guaiacol	10 (c)	Sweet. smoke	4.6	108 ± 22	nd	nd
Eugenol	6 (c)	Spices. clove. honey	4.5	nd	142 ± 62	42 ± 13
Ethyl phenol				nd	nd	nd
4 vinyl guaiacol	40 (a)	Spices. curry	5	363 ± 151	248 ± 54	218 ± 24
4 Hydroxy methyl acetophenone				nd	163 ± 42	nd
Siringol				299 ± 80		148 ± 0
TOTAL				770 ± 231	553 ± 158	408 ± 37
**LACTONES**						
Y-butyrolactone	35 (c)	Sweat. toasted	4	175 ± 116	96 ± 37	174 ± 10
Cis methyl 4 octanolide	67		4	nd	nd	89 ± 3
TOTAL				175 ± 116	96 ± 37	262 ± 13
**TERPENS**						
Terpineol	110		2	73 ± 1	50 ± 0	30 ± 12
TOTAL						
**ACIDS**						
Isobutyric acid	2300 (b)	Rancid. butter. cheese	6	166 ± 138	93 ±31	212 ± 24
Butyric acid	173 (b)	Rancid. cheese. sweat	6	115 ± 50	83 ± 14	85 ± 3
(3 methyl butanoic) isovaleric acid	33 (c)	Sweet. acid	4.6	244 ± 58	269 ± 105	434 ± 10
Hexanoic acid	420 (b)	Sweet	6	2366 ± 96	2161 ± 67	2159 ± 115
Octanoic acid	500 (c)	Sweet. cheese	6	4716 ± 372	4372 ± 1098	3922 ± 149
Decanoic acid	1000 (b)	Rancid. fat	6	1178 ± 10	1344 ± 13	1278 ± 121
TOTAL				8785 ± 725	8322 ± 1328	8090 ± 422

*The pure culture of S. cerevisiae was used as control*.

*Values expressed in μg/L are the mean of two injections. The standard deviation values (±) are indicated. n.d. not detectable*.

a *(a) Guth (1997); (b) Etievant (1991); (c) Ferreira et al. (2000)*.

b *Odorant series: 1 = Fruity; 2 = Floral; 3 = Green; 4 = Sweet; 5 = Spicy; 6 = Fatty; 7 = Others*.

Because alcohols are also important compounds influencing wine aroma, it is important to highlight that wine produced by co-inoculation contained higher 1-propanol, 1-butanol and isoamyl alcohols concentrations. Among identified alcohols, 2-phenylethanol was the second most abundant alcohol at concentrations higher than its threshold in all wines, contributing with fine rose's notes to wine aroma. In wines analyzed, we observed differences in α-terpineol concentration; in fact, this compound was identified and quantified in a major concentration in co-inoculated wine. Within the family of fatty acids, isobutyric, isovaleric, hexanoic, hexanoic, octanoic and decanoic acids were notable for their high concentrations in all wines and have been described with fruity, cheese, fatty, and rancid notes (Rocha et al., 2004).

The two mixed fermentations show an overall more complex aromatic profile than the pure culture of *S. cerevisiae*. Its sweet, spicy, floral odorant notes characterized the sequential mixed fermentation. Simultaneous fermentation of *H. uvarum* and *S. cerevisiae* was characterized by fruity and sweet aroma descriptors (Table 2).

### Industrial vinification

These large-scale experiments were conducted in a winery cellar of Salento by simultaneous inoculation, with the selected mixed starter *H. uvarum* ITEM8795*/S. cerevisiae* ITEM6920, of 7 tons of Negroamaro must. The data corresponding to the fermentation performance of the two isolates used and their ability to dominate the fermentation indicated that these two autochthonous yeast strains possess the fundamental properties required for starter cultures, in fact, the fermentations progressed regularly and sugar depletion was accomplished in 10 days.

**Figure 4:**
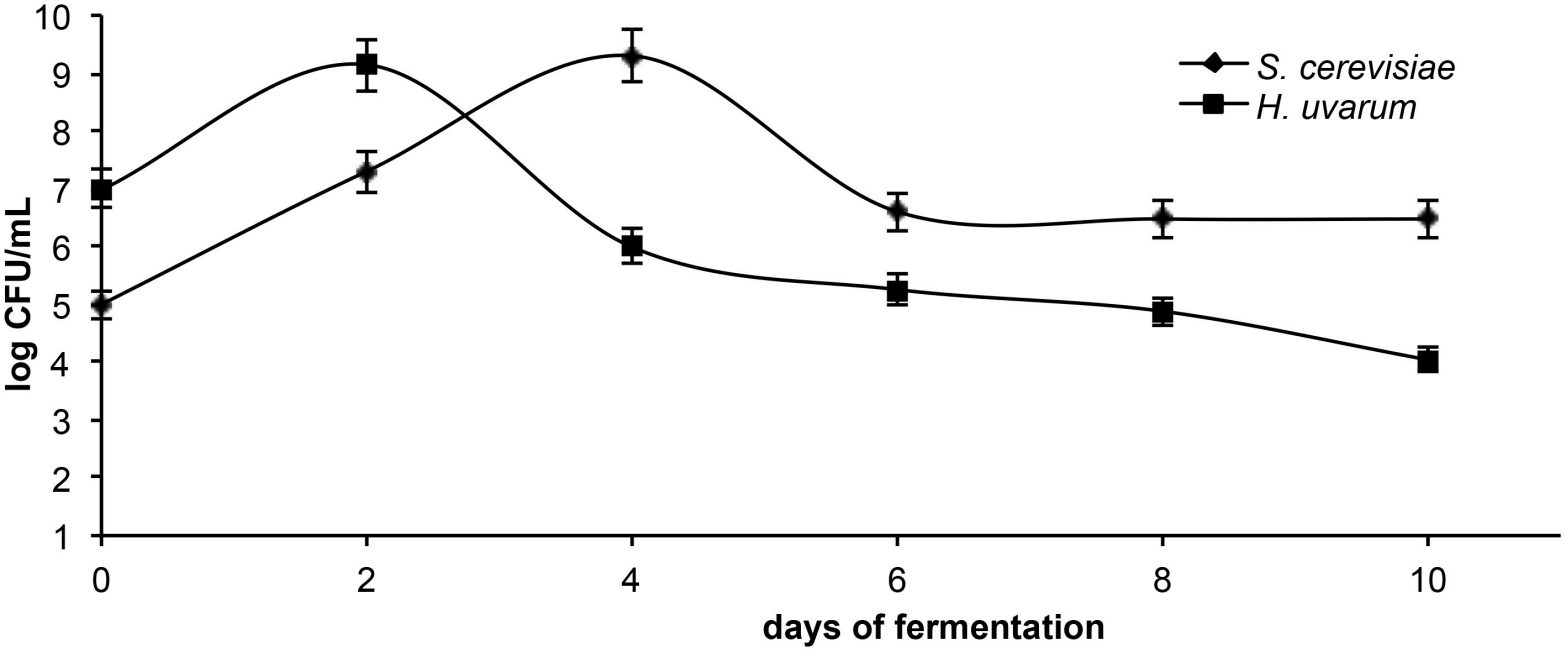
**Viable cell counts of the two inoculated yeast species throughout the industrial vinification**.

Viable cells counts of the two yeast species throughout the fermentation are shown in Figure 4. *H. uvarum* dominated the early stages of fermentation and its population reached the maximum (10^9^ CFU/mL) at the 2nd day; then gradually decreased to 10^4^ CFU/mL and keep stable until the end of the fermentation period. *S. cerevisiae* dominate the fermentation from day 4th, when it reached a concentration of 10^9^ CFU/mL; then slightly decreased to 10^6^ CFU/mL and ultimate the fermentation by day 8.

**Figure 5:**
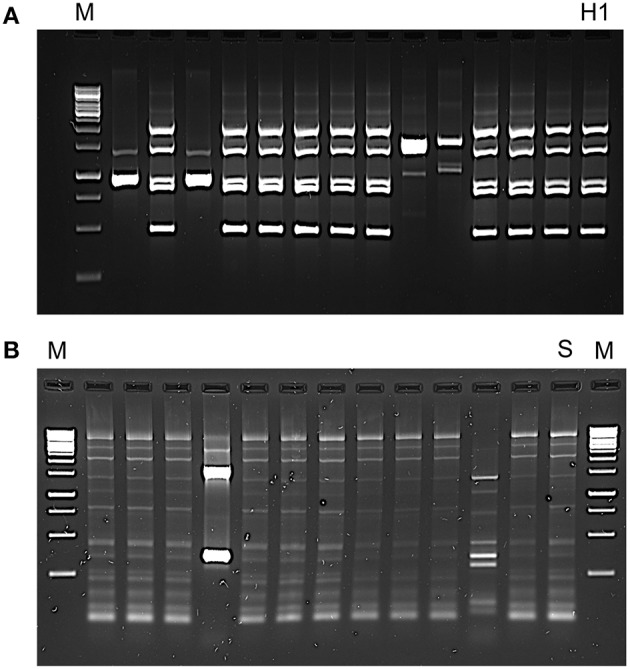
**Electrophoretic profiles patterns of (A) RAPD analysis with primer RM13 of *Hanseniaspora uvarum* randomly isolated from the large scale fermentation**. The strain-specific profile of the 8795 strain is reported (H1); **(B)** interdelta region patterns obtained from *Saccharomyces cerevisiae* randomly isolated at the end of the large scale fermentation. The strain-specific profile for the 6920 strain is reported (S). Molecular marker (M): Thermo Scientific GeneRuler 1 Kb DNA Ladder.

The dominance of the inoculated strains was confirmed by molecular analysis. The electrophoresis patterns of green colonies isolated on WLN agar at middle fermentation stage are shown in Figure 5A. It can be observed that 9 out of 13 isolates have the same profile than that of the inoculated starter *H. uvarum* ITEM 8795 (H1), thus indicating that this strain got the upper hand of indigenous non-*Saccharomyces* strains. Likewise, the electrophoresis patterns of pale cream colonies isolated on WLN agar at the end of the fermentation are shown in Figure 5B. In this case, the 83% of isolates exhibit an identical profile to the one of the inoculated starter *S. cerevisiae* ITEM 6920, it being the evidence that the above starter was able to dominate the final steps of the AF. The results of chemical analysis of the wine obtained by co-fermentation *H. uvarum*/*S. cerevisiae* are shown in Table 3, in comparison to the same must fermented with the commercial starter in use in the winery. The total acidity was higher in must fermented by mixed starter (5.84 g/L), while volatile acidity was lower (0.43 g/L) than in must fermented with the commercial *S. cerevisiae* (5.49 and 0.45 g/L, respectively). Both starters were able to metabolize completely sugars. Furthermore, the mixed starter showed a lower alcohol content (13.99 mL/100 mL).

**Table 3:** **Analysis of final wine obtained by cofermentation of *H. uvarum* and *S. cerevisiae* in comparison to the same must fermented with the commercial starter in use in the industrial vinification**.

**Compound**	**Cofermentation *H. uvarum*/*S. cerevisiae***	**Commercial starter**
Alcohol (mL/100 mL)	13.99 ± 0.003	14.03 ± 0.01
Residual sugars (g/L)	n.d.	n.d.
Total acidity (g/L)	5.84 ± 0.067	5.49 ± 0.028
Volatile acidity (g/L)	0.43 ± 0.005	0.45 ± 0.003
pH	3.48 ± 0.009	3.44 ± 0.003
Malic acid (g/L)	1.1 ± 0.008	0.96 ± 0.005
Lactic acid (g/L)	0.18 ± 0.034	0.17 ± 0.023
Tartaric acid (g/L)	2.34 ± 0.105	1.89 ± 0.021
Citric acid (g/L)	0.45 ± 0.011	0.43 ± 0.02
Density (g/mL)	0.99093 ± 0.00003	0.99025 ± 0.000043
Dry matter (g/L)	22.79 ± 0.112	21.11 ± 0.111
Glycerol (g/L)	7.07 ± 0.014	7.01 ± 0.038
Methanol (mL/100 mL)	n.d.	n.d
Total polyphenols (mg/L)	547 ± 92	671 ± 25
Anthocyanins (mg/L)	410 ± 71	180 ± 22
Absorbance at 420	0.88 ± 0.001	0.81 ± 0.028
Absorbance at 520	0.97 ± 0.001	1.11 ± 0.031
Absorbance at 620	0.41 ± 0.001	0.23 ± 0.032

*Values are the mean of three injections; the standard deviation values (±) are indicated; n.d. not detectable*.

### Comparation of selected volatile compounds concentration in wine produced in lab-, pilot-, and industrial scale

The influence of the mixed starter *H. uvarum/S. cerevisiae*, used to produce Negroamaro wine in laboratory-, pilot-, and industrial scale, on the organoleptic quality of wines was assessed by comparing the concentrations of specific volatile compounds, produced by yeast metabolism (Table 4). Each single analyzed compound, chosen between different esters, acids, alcohols, terpenes, and aldehydes, showed comparable concentration in the wines produced by the three vinifications.

**Table 4:** **Concentration of selected volatile compounds in wines obtained with the mixed starter *H. uvarum*/*S. cerevisiae* used in simultaneous inoculum**.

	**Lab-scale**		**Pilot-scale**		**Industrial-scale**	
	**Control**	**H1+S**	**Control**	**H1+S**	**Control**	**H1+S**
**ESTERS**						
Isoamyl acetate	369.35	2330.00	2234.67	2239.18	312.05	2596.76
Ethyl hexanoate	434.51	510.00	510.49	560.85	433.73	547.57
Ethyl octanoate	371.69	604.91	406.42	573.12	476.00	661.15
3-Hydroxy-ethyl butanoate	53.79	69.78	52.23	65.35	51.41	67.74
Ethyl decanoate	183.24	230.00	188.19	252.18	234.87	229.38
Phenylethyl acetate	413.57	620.00	516.66	695.84	493.73	649.92
Ethyl acetate (mg/L)	42.11	84.78	22.07	92.04	25.05	87.04
**ALCOHOLS**						
Isoamyl alcohols	547.85	750.00	554.01	767.61	680.33	801.87
Phenylethylalcohol	11,480.03	11,994.45	13,760.43	12,962.32	10,555.51	11,716.07
**ACIDS**						
Hexanoic acid	2088.15	2246.68	2159.34	2366.45	2200.32	2246.68
Octanoic acid	3869.21	4574.65	3921.89	4716.22	3722.84	4574.65
**TERPENS**						
Terpineol	54.13	66.50	50.40	72.80	57.15	68.80
**KETONS/ALDEHYDES**						
Acetoin (mg/L)	4.11	11.24	7.65	11.85	6.05	11.34
Acetaldehyde (mg/L)	5.04	25.05	6.05	28.00	5.11	24.11

*The pure culture of S. cerevisiae was used as control*.

When compared to the wines produced by using the *S. cerevisiae* as starter, the three wines produced by inoculation of the mixed starter showed an increment of acetate esters (ethyl acetate, isoamyl acetate, and phenylacetate) and fatty acids esters (ethyl hexanoate, ethyl octanoate, and ethyl decanoate). Esters is one of the large groups of volatiles found in wines. These compounds are important in young wine aroma and are among key compounds in the fruity flavors of wines (Rapp and Mandery, 1986). Ethyl acetate, in particular, adds complexity to the aroma of wine, with fruity notes at concentrations lower than 150 mg/L, while at higher concentrations it can donate a sour, vinegary off-odor. Its higher concentration was found in H1+S industrial scale (87.04 mg/L).

Regarding alcohols, in particular isoamylalcohols and 2-phenylethanol were determined in the analyzed wines and they resulted to be quantitatively the most representative compounds in this group, showing a higher concentrations of these molecules when compared to the wines produced by the *S. cerevisiae* starter. Isoamylalcohols can have both positive and negative impacts on wine aroma. In fact alcohols concentrations exceeding 400 mg/L can have a detrimental effect (Rapp and Versini, 1991; Romano et al., 1997), whereas lower concentrations impart positive fruity characters (Lambrechts and Pretorius, 2000; Saurina, 2010). In our sample the concentrations detected were below this threshold. However, 2-phenylethanol was the second most abundant alcohol at concentrations higher than its threshold (10 mg/L), contributing with fine rose's notes to wine aroma.

## Discussion

The utilization of non-*Saccharomyces* starters together with *Saccharomyces cerevisiae* in grape must fermentations has been investigated by Zironi and coworkers since 1993. The addition of yeasts belonging to non-*Saccharomyces* species as part of formulations of mixed starters, together with *S. cerevisiae*, has recently been indicated as a way to mimic the biotechnological potential associated with spontaneous fermentations to improve the quality of the wine (Rojas et al., 2001; Romano et al., 2003; Ciani et al., 2010).

Several non-*Saccharomyces* species, such as *H. uvarum, Zygosaccharomyces bailii, Lachancea thermotolerans, Candida cantarelli*, and *C. zemplinina* have been studied thus far in mixed fermentations with the scope of adding peculiar features to the wine (Toro and Vazquez, 2002; Ciani et al., 2006; Comitini et al., 2011; Suzzi et al., 2012; Gobbi et al., 2013; Garavaglia et al., 2015). In fact, a current trend in the wine market is to develop unique products, thus the mixed starter could be a good approach to give a special flavor and improve the quality of wines from both the organoleptic and microbiological point of view (Zironi et al., 1993; Mingorance-Cazorla et al., 2003; Capozzi et al., 2015; Lu et al., 2015; Masneuf-Pomarede et al., 2016). Moreover, in the contexst of the oenological production of Southern Italy (and other similar climates) denoted by high alcohol content and high total acidity, the preliminary utilization of a non-*Saccharomyces* starter (fructophylic and able to produce low amounts of acetic acid), might be an interesting approach in order to consume sugars in the early stage of fermentation, thus reducing the impact of osmotic stress for the *S. cerevisiae* starter (Rantsiou et al., 2012; Tofalo et al., 2012).

In the present investigation, we evaluated the fermentation performance of a culture of non-*Saccharomyces* yeasts belonging to the oenological species *H. uvarum* in micro-fermentation and, thereafter, in fermentations on pilot and industrial scale, conducted in mixed fermentations with yeasts belonging the species *S. cerevisiae*. These two different cultures were inoculated simultaneously or sequentially and the fermentation dynamics were studied in both fermentations. From the results of this series of tests, we obtained useful information on the kinetics of growth and fermentation activity, supported by analytical data of fermented musts and final wines.

In micro-fermentation trials, the presence of *S. cerevisiae* stimulated the persistence of the non-*Saccharomyces* strains during the fermentation process, in accordance with previous studies (Ciani et al., 2006; Mendoza et al., 2007; Mendoza and Farías, 2010), and this effect was more relevant in the sequential fermentations. Indeed, the three *H. uvarum* strains stayed viable, at significant high concentration levels of about 10^4^–10^6^ CFU/mL until the end of the fermentation even with an alcohol content of about 12% (v/v). On the other hand, in the simultaneous inoculation, the presence of the non-*Saccharomyces* strains since the early stages of fermentation seems to affect the cell growth and biomass production of *S. cerevisiae* probably due to the competition for nutrients (Mendoza et al., 2007; Domizio et al., 2011; Suzzi et al., 2012). However, the interactions between the two species during grape must/wine fermentation should be further studied and deepened. In fact, the knowledge about the metabolic interactions between *S. cerevisiae* and non-*Saccharomyces* strains in winemaking is still limited (Wang et al., 2015). Nevertheless, the fermentation rates of the mixed fermentation were comparable to that of the *S. cerevisiae* pure culture. Regardless the biomass production or fermentation rates, all the mixed cultures reached the completion but produced lower concentrations of ethanol than the pure culture of *S. cerevisiae* in accordance with previous studies (Mendoza and Farías, 2010; Mendoza et al., 2011).

The fermentations on a laboratory scale carried on regularly and the analysis of the corresponding fermented musts have not revealed the presence of compounds with possible negative impact to a level that will exceed the threshold of sensory perception. On the contrary, wines obtained with the association *H. uvarum*/*S. cerevisiae* showed some interesting characters. In fact, the evidence obtained during this investigation confirm previous data indicating that the combination and the interaction between the starter cultures of *S. cerevisiae* and non-*Saccharomyces* species has led to a reduction of acetic acid, even at concentrations lower than those produced by the pure culture of *S. cerevisiae* (Ciani et al., 2006; Mendoza and Farías, 2010; Domizio et al., 2011).

Several studies on the use of associated *S. cerevisiae* and non-*Saccharomyces* yeasts have highlighted many of the positive effects produced in these mixed fermentations such as the increasing in isoamyl acetate and 2-phenyl acetate (Moreira et al., 2008; Andorrà et al., 2010) or glycerol (Ciani and Ferraro, 1996) content in wine. Indeed, in the trial H1+S_sm, it was possible to note an increase of glycerol as well as of some volatile compounds, such as esters and aliphatic higher alcohols, as previously reported (Garde-Cerdán and Ancín-Azpilicueta, 2006). However, the impact of glycerol on the wine quality is still under discussion (Marchal et al., 2011).

These results were further confirmed in a pilot-scale vinification using a *H. uvarum* strain (ITEM 8795) in combination with *S. cerevisiae* ITEM 6920. The wines produced using two different strategies of inoculation (simultaneous and sequential) of the *H. uvarum*/*S. cerevisiae* starter were compared with that obtained after inoculation of a pure culture of *S. cerevisiae*, mainly focusing on their aromatic profile. It was also observed a different use of sugars in the tests in co-inoculation with *H. uvarum*. In fact, this fructophilic yeast interacts positively with the strain of *S. cerevisiae*, which is glucophilic, with the result of a more rapid utilization of the sugars (Ciani and Fatichenti, 1999). *H. uvarum* ITEM 8795, in simultaneous and sequential cultures, showed the maximal cell concentration after 2 days and then they die but remained in countable numbers until the end of the fermentation. This behavior of the apiculate yeast is in agreement with data reported in literature, which indicate that non-*Saccharomyces* yeasts dominate during the first 3–4 days of fermentations up to an ethanol concentration of about 4–7% (v/v) and then they start the phase of death (Fleet and Heard, 1993; Fleet, 2003). Moreover, it has been demonstrated that non-*Saccharomyces* yeasts kept their viability for longer period in composite cultures with *S. cerevisiae* (Ciani et al., 2006; Mendoza et al., 2007). The estimation of some of the principal volatile compounds confirmed that the *H. uvarum* ITEM 8975 did not form high amounts of ethyl acetate in mixed cultures (De Benedictis et al., 2011). However, in mixed cultures, the concentration of ethyl acetate produced are likely to contribute to the fruity notes and add to the general complexity to the produced wine (Ciani et al., 2006). The *H. uvarum* ITEM 8975 confirmed to be an acetoin low-producer even in multi-starter fermentations, it being this compound probably also consumed by the vigorously fermenting *S. cerevisiae* starter strain (Romano et al., 2003).

The amounts of acetaldehyde, a relevant secondary product of fermentation (Romano et al., 1997), did not appear to be negatively influenced by mixed cultures of *H. uvarum*, with a behavior similar to that described by Ciani et al. (2006) during the studies of lab-scale *H. uvarum* multi-started fermentations.

Ethyl esters concentrations are influenced by yeast strain, fermentation temperature, aeration degree and sugar content. Both ethyl esters and acetate esters have a key importance in the whole wine aroma impressing a positive contribution by distinct sensory notes: sweet-fruity, grape-like odor, sweet-balsamic (Rapp, 1990; Swiegers and Pretorius, 2005). Indeed, wine yeasts such as *Hanseniaspora* spp. in mixed fermentations with *S. cerevisiae*, have improved the formation of esters with a positive sensorial impact, as well as the reduction of volatile acidity production (Rojas et al., 2003; Moreira et al., 2005; Medina et al., 2013). Chemical analysis of the wines produced using the mixed cultures *H. uvarum*/*S. cerevisiae* clearly differ from wine produced with the solo *S. cerevisiae*. Both mixed fermentations led to a higher content of esters such as 2-phenylethyl acetate, which is in agreement with previous studies conducted with *H. vineae* (Viana et al., 2011; Medina et al., 2013) and *H. guilliermondii* (Rojas et al., 2003; Moreira et al., 2011). This compound contributes to the rose, honey, fruity and flower aromas of wines (Swiegers et al., 2005). Likewise, 2-phenylethanol contributes with a floral (rose) aroma in the final wine (Swiegers et al., 2005) though, an excess in higher alcohols concentrations in wine would bring a strong, pungent smell and taste (Moreira et al., 2011). In our study, the use of the apiculate yeast *H. uvarum* in mixed starter culture with *S. cerevisiae* decreased the total higher alcohol content and resulted in a concentration of 2-phenylethyl alcohol just above its sensory threshold (Moreira et al., 2008; Medina et al., 2013). Mixed fermentations also resulted in decreases in isovaleric acid and increases in hexanoic, octanoic acid and ethyl octanoate. Moreover, the presence of higher levels of decanoic acid and ethyl decanoate was correlated with greater rates of cell lysis, which could contribute to the tropical fruit aroma, texture and body of wines (Medina et al., 2013). On the basis of the above findings, we can say that co-inoculation represents an alternative approach in commercial winemaking and its success strongly depends on the selection of suitable yeast strains. In this study carried out at industrial level, the use of selected yeasts provides good results in terms of lack of wine alterations. The scale-up of mixed fermentation, for the first time, to an industrial level was the key step to validate the results obtained in the laboratory and in pilot-scale. The winemaking process has largely confirmed both the evolution of the cultures inoculated and the analytical characteristics of wines given by the strains of *H. uvarum* and *S. cerevisiae* used for the fermentation. The results obtained were supported by the fact that both inoculated strains were dominant on indigenous microflora and, thus, they have certainly conducted the fermentative process. The data achieved during the present investigation confirmed the concept that oenological non-*Saccharomyces* yeasts represent a resource of great value for the winemaking industry. Indeed, the obtained results indicated the *H. uvarum* strain ITEM 8795 can be used in association with *S. cerevisiae* starter cultures in the in the winemaking conditions typical of Southern Italy (Puglia) wine production. The here-described multi-starter fermentation was able to enhance the quality, improve the aromatic profile and reduce the effect of the undesired characters of the final Negroamaro wine.

## Author contributions

All authors significantly contributed to this paper. FG and GS conceived and designed the experiments; MTr, MTu, and VC performed the experiments; FG, GS, MTr, MTu, GM, and VC analyzed the data; FG was responsible for manuscript preparation and submission; FG, GS, MTr, MTu, GM, and VC reviewed the paper.

### Conflict of interest statement

The authors declare that the research was conducted in the absence of any commercial or financial relationships that could be construed as a potential conflict of interest.
